# Polymyalgia Rheumatica (PMR) Lacking Shoulder Pain Following COVID-19 Vaccination

**DOI:** 10.7759/cureus.34714

**Published:** 2023-02-07

**Authors:** Kotaro Haruna, Shogo Shirota, Hiroaki Nishioka

**Affiliations:** 1 General Internal Medicine, Kobe City Medical Center General Hospital, Hyogo, JPN

**Keywords:** pmr, covid-19, fdg-pet, covid-19 vaccination, polymyalgia rheumatica

## Abstract

This case highlights a 77-year-old woman with polymyalgia rheumatica (PMR) following coronavirus disease 2019 (COVID-19) vaccination without shoulder pain and its characteristic positron emission tomography-computed tomography (PET-CT) findings. Some cases of PMR following COVID-19 vaccination have been reported. However, patients without shoulder pain or PET-CT findings without uptake at the shoulders are not described. Our case suggests that PMR should be considered in the differential diagnosis of patients with neck pain following COVID-19 vaccination, even if they lack shoulder pain.

## Introduction

Polymyalgia rheumatica (PMR) is a common inflammatory rheumatic disorder that affects older adults. It is characterized by pain and stiffness of the shoulders and pelvic girdle [1]. Although the etiology and the pathogenesis of PMR remain unknown, environmental triggers such as infection and vaccination are thought to play a role. The COVID-19 pandemic resulted in over six million deaths worldwide, with older adults accounting for the majority of the deaths [2-3]. Two messenger ribonucleic acid (mRNA) based vaccines were rapidly developed by Pfizer/BioNTech and Moderna to prevent COVID-19. Large clinical studies have shown that these vaccines are safe and effective in preventing infection and reducing disease severity and death. However, there have been some reports of immune-mediated adverse events, such as vasculitis, arthritis, myasthenia gravis, and idiopathic myocarditis, after COVID-19 vaccination [4]. Herein, we report the case of an older patient who developed PMR lacking shoulder pain following the administration of a COVID-19 mRNA vaccine.

## Case presentation

**Figure 1:**
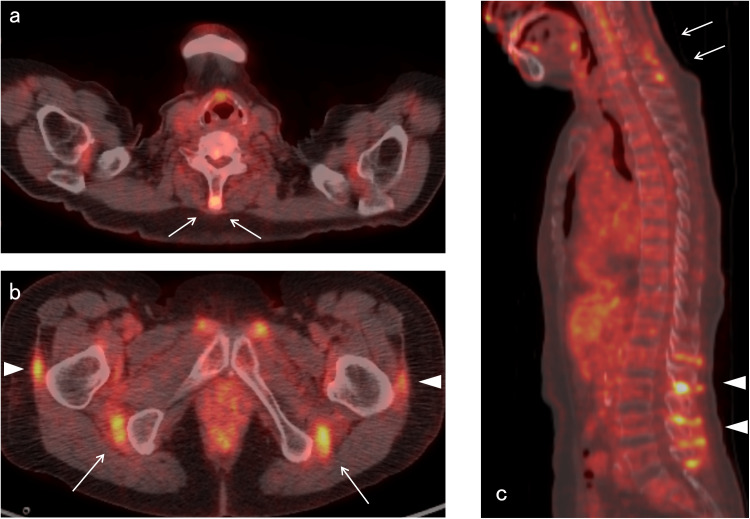
FDG PET-CT. FDG PET-CT, fluorodeoxyglucose-positron emission tomography FDG PET-CT reveals (a) increased FDG uptake at the cervical interspinous bursae (arrows), (b) the ischial tuberosities (arrows) and the greater trochanters (arrowheads), and (c) the interspinous bursae of the cervical (arrows) and lumbar regions (arrowheads).

A 77-year-old Japanese woman with no past medical history presented with a four-week history of neck pain. Two days after receiving the second COVID-19 mRNA vaccine [(BNT162b2, Pfizer (Pfizer Inc. in, New York, NY)/BioNTech (Mainz, Germany)], she developed myalgia at the posterior and lateral neck, which worsened in the morning. Her symptoms persisted for four weeks; low-grade fever and tiredness in the thighs accompanied them; however, she did not have shoulder pain. Physical examination revealed tenderness at the cervical spinous processes, the sternocleidomastoid muscles, and the greater trochanters, not at the shoulders or hips. The range of motion of the shoulders was not limited. The painful arc test was negative. Muscle strengths were normal. Laboratory tests showed a C-reactive protein (CRP) level of 4.32 mg/dL, an erythrocyte sedimentation rate (ESR) of 100 mm/h, and a normal level of creatine kinase. The test results for rheumatoid factor, anti-cyclic citrullinated peptide antibodies, and the other autoantibodies related to myopathy were negative. The blood culture did not grow any bacteria. Ultrasonography of the shoulders detected no abnormal findings. Positron emission tomography-computed tomography (PET-CT) revealed increased 18F-fluorodeoxyglucose (FDG) uptake at the cervical and lumbar interspinous bursae, ischial tuberosities, trochanteric areas, and hips, but not at the shoulders (Figure 1). The patient met the classification criteria for PMR (Table 1) [5-6], and the PET-CT findings were compatible. We initiated the administration of prednisolone at a dose of 20 mg/day. Her symptoms markedly improved over several days, and her CRP level decreased to normal.

**Table 1:** Two PMR classification criteria satisfied in this case. PMR, polymyalgia rheumatica; ESR, erythrocyte sedimentation rate; RF, rheumatoid factor; ACPA, anti-cyclic citrullinated peptide antibodies

Chuang’s criteria [5]	Haeley’s criteria [6]
Age ≧ 50 years	Age ≧ 50 years
ESR > 40 mm/h	Absence of RF or ACPA
Exclusion of other diseases except for giant cell arteritis	≧ 3 of the followings:
Bilateral aching and stiffness > 1 month in ≧ 2 of:	Pain in the neck, shoulders, or pelvic girdle
Neck or torso	Elevated ESR
Shoulders or proximal regions of the arms	Rapid response to prednisone ≦ 20 mg/day
Hips or proximal aspects of the thighs	Marked morning stiffness > 1 h

## Discussion

A handful of cases of PMR following COVID-19 vaccination have been reported [7-9], and these patients presented with shoulder pain. Shoulder pain is a typical symptom of PMR and is reported to be found in over 90% of patients; however, some cases of PMR lacking shoulder symptoms have been reported [10]. In addition, some classification criteria do not necessarily require shoulder pain, although it is part of the items [5-6, 11]. Recent research on the diagnostic value of FDG-PET/CT in PMR reported that ischiatic and interspinous bursae uptake on PET are suggestive of PMR, and three or more locations of 17 hotspots with increased uptake are associated with the diagnosis of PMR [12]. A meta-analysis revealed the negative likelihood ratio of uptake at the shoulder was found to be 0.31, which is unremarkable [13]. Our patient may be the first case of PMR lacking shoulder pain following COVID-19 vaccination. PMR without shoulder pain is uncommon but should not be overlooked.

The causal relationship between PMR and COVID-19 vaccination remains to be elucidated. The increased expression of toll-like receptor 7 (TLR7), which recognizes viral single-strand RNA, and potential saturation of TLR7 signaling have been observed in the peripheral B cells, T cells, and monocytes of patients with PMR [14]. A recent study of human whole blood demonstrated strong activation of TLR signaling by the administration of BNT162b2 vaccine, especially after the second dose [15]. One hypothesis is that the activation of TLR signaling by BNT162b2 possibly acts as a trigger for PMR [8]. BNT162b2 vaccine also contains polyethylene glycol (PEG)-conjugated lipid shells encapsulating the mRNA. PEG has been reported to cause acute infusion reactions and has been speculated to be the major cause of anaphylaxis to BNT162b2 [16]; however, to our knowledge, reports concerning PEG and PMR have been rarely reported. This case cannot conclusively determine whether or not there is a causal relationship between PMR and COVID-19 vaccination. We cannot exclude the possibility that the patient developed PMR coincidentally. Further studies involving surveillance of similar cases are needed to clarify these issues.

## Conclusions

This case demonstrates that PMR lacking shoulder pain developed following COVID-19 vaccination. PMR should be considered in the differential diagnosis of patients with neck pain and raised markers of inflammation following COVID-19 vaccination, even if they lack shoulder pain. As this is a rare adverse event of the COVID-19 vaccine and COVID-19 poses a much greater risk than PMR, the vaccine’s benefits should not be dismissed.

## References

[1] Buttgereit F, Matteson EL, Dejaco C (2020). Polymyalgia rheumatica and giant cell arteritis. JAMA.

[2] (2023). Johns Hopkins University and Medicine Coronavirus Resource Center. https://coronavirus.jhu.edu.

[3] Wiersinga WJ, Rhodes A, Cheng AC, Peacock SJ, Prescott HC (2020). Pathophysiology, transmission, diagnosis, and treatment of coronavirus disease 2019 (COVID-19): a review. JAMA.

[4] Watad A, De Marco G, Mahajna H (2021). Immune-mediated disease flares or new-onset disease in 27 subjects following mRNA/DNA SARS-CoV-2 vaccination. Vaccines (Basel).

[5] Chuang TY, Hunder GG, Ilstrup DM, Kurland LT (1982). Polymyalgia rheumatica: a 10-year epidemiologic and clinical study. Ann Intern Med.

[6] Healey LA (1984). Long-term follow-up of polymyalgia rheumatica: evidence for synovitis. Semin Arthritis Rheum.

[7] Ottaviani S, Juge PA, Forien M, Ebstein E, Palazzo E, Dieudé P (2022). Polymyalgia rheumatica following COVID-19 vaccination: a case-series of ten patients. Joint Bone Spine.

[8] Manzo C, Castagna A, Isetta M (2022). Polymyalgia rheumatica and polymyalgia-like syndromes as adverse events following COVID-19 vaccines: working notes from a narrative review of published literature. Reumatologia.

[9] Osada A, Sakuragi C, Toya C, Mitsuo A (2022). New-onset polymyalgia rheumatica following the administration of the Pfizer-BioNTech COVID-19 vaccine. Intern Med.

[10] Fitzcharles MA, Esdaile JM (1990). Atypical presentations of polymyalgia rheumatica. Arthritis Rheum.

[11] Nobunaga M, Yoshioka K, Yasuda M, Shingu M (1989). Clinical studies of polymyalgia rheumatica. A proposal of diagnostic criteria. Jpn J Med.

[12] Sondag M, Guillot X, Verhoeven F, Blagosklonov O, Prati C, Boulahdour H, Wendling D (2016). Utility of 18F-fluoro-dexoxyglucose positron emission tomography for the diagnosis of polymyalgia rheumatica: a controlled study. Rheumatology (Oxford).

[13] van der Geest KS, Treglia G, Glaudemans AW, Brouwer E, Jamar F, Slart RH, Gheysens O (2021). Diagnostic value of [18F]FDG-PET/CT in polymyalgia rheumatica: a systematic review and meta-analysis. Eur J Nucl Med Mol Imaging.

[14] Álvarez Rodríguez L, López-Hoyos M, Mata C (2011). Expression and function of toll-like receptors in peripheral blood mononuclear cells of patients with polymyalgia rheumatica and giant cell arteritis. Ann Rheum Dis.

[15] Arunachalam PS, Scott MK, Hagan T (2021). Systems vaccinology of the BNT162b2 mRNA vaccine in humans. Nature.

[16] Sellaturay P, Nasser S, Islam S, Gurugama P, Ewan PW (2021). Polyethylene glycol (PEG) is a cause of anaphylaxis to the Pfizer/BioNTech mRNA COVID-19 vaccine. Clin Exp Allergy.

